# Associations between fitness, movement behaviors, and immediate post-exercise blood pressure in older adults: A network perspective

**DOI:** 10.1371/journal.pone.0329280

**Published:** 2025-07-30

**Authors:** Gabriel Costa Souto, Ana Clara Cassimiro Nunes, Rodrigo Alberto Vieira Browne, Yuri Alberto Freire, Paulo Felipe Ribeiro Bandeira, Eduardo Caldas Costa

**Affiliations:** 1 Graduate Program in Health Sciences, Federal University of Rio Grande do Norte, Natal, Brazil; 2 Department of Physical Education, ExCE Research Group, Federal University of Rio Grande do Norte, Natal, Brazil; 3 Graduate Program in Physical Education, Federal University of Vale do São Francisco, Petrolina, Brazil; 4 Gradute Program in Physical Education, Regional University of Cariri, Crato, Brazil,; 5 Graduate Program in Physical Education, Catholic University of Brasília, Brasília, Brazil; Japanese Academy of Health and Practice, JAPAN

## Abstract

Understanding the associated factors of immediate post-exercise blood pressure, a proxy for exercise blood pressure, is relevant for improving cardiovascular risk assessment and guiding interventions. This study investigated associations between fitness and movement behavior components with immediate post-exercise blood pressure in older adults using network analysis. In this cross-sectional study, 237 older adults (66 ± 5 years), without known cardiovascular disease were included. Blood pressure was measured before and immediately after a 3-minute moderate walking test (5 METs). Moderate-to-vigorous physical activity and sedentary time were assessed using hip-worn accelerometers for one week. Proxies for fitness were: six-minute walking test, handgrip strength, and 30-second sit-to-stand test. Network analysis, a multivariate statistical approach which captures interrelations among multiple variables within a system, was used to examine direct and indirect associations between fitness, movement behaviors, and post-exercise blood pressure. Lower handgrip strength (weight: −0.136) and 30-second sit-to-stand (weight: −0.106) performances were directly associated with greater immediate post-exercise blood pressure, indicating that reduced muscular strength is linked to greater cardiovascular load after exertion. Lower handgrip strength (weight: −0.176) and reduced physical activity (weight: −0.128) were directly associated with higher resting BP, which in turn had the strongest direct association with immediate post-exercise blood pressure (weight: 0.581), suggesting an indirect pathway through resting blood pressure. Centrality indicators suggested six-minute walking test, 30-second sit-to-stand test, and sedentary time as potential interventions targets, moderate-to-vigorous physical activity as a central hub within network, and handgrip strength as a highly connected node. Our findings suggest a complex interplay – both direct and indirect (via resting blood pressure) – between fitness, movement behaviors, and immediate post-exercise blood pressure in older adults. Increasing moderate-to-vigorous physical activity, cardiorespiratory fitness, and muscle strength, along with reducing sedentary time, may be potential targets for attenuating immediate post-exercise blood pressure in this age group.

## Introduction

Elevated blood pressure (BP) is a well-established and independent risk factor for cardiovascular disease (CVD) and mortality, with decades of epidemiological and clinical research supporting risk stratification and treatment guidance [1,2]. However, resting BP may not fully capture the complexity of cardiovascular regulation under physiological stress. Exercise blood pressure (BP), defined as the BP response to physical effort, has emerged as a complementary marker capable of identifying cardiovascular abnormalities, such as a hypertensive response to exercise [3]. Although traditionally identified at maximal effort during exercise testing, hypertensive response to exercise (also termed exaggerated exercise BP, hypertensive response to exercise, exercise-induced hypertension, or exercise hypertension) at submaximal efforts has been associated with an increased risk of CVD and mortality, regardless of resting BP [4,5]. For example, exercise BP ≥ 170 mmHg at stage 2 of a Bruce protocol was associated with a 33% adjusted increased risk of adverse CVD-related events and mortality [6]. In addition, a hypertensive response to exercise may indicate underlying cardiovascular impairments such as vascular dysfunction [7], increased arterial stiffness [8], or impaired baroreflex sensitivity [9].

Several factors are associated with exercise BP, such as resting BP, age, sex, presence of CVD, and other traditional cardiometabolic risk factors [5]. In addition, some studies have shown that exercise BP seems to be attenuated in individuals with higher fitness levels, including cardiorespiratory fitness and muscle strength [10,11], when exercising at a fixed submaximal external load (i.e., walking at a given speed and grade). Recently, one study [12] explored the direct and indirect association of fitness, body composition, and CVD risk factors on exercise BP at submaximal effort in middle-aged individuals. Cardiorespiratory fitness, but not muscle strength, along with body composition, and traditional CVD risk factors were associated with exercise BP, mainly indirectly via resting BP. However, movement behaviors (sedentary time and physical activity) were not included in the analysis. Since fitness is bidirectionally associated with physical activity levels, more active individuals may exhibit lower exercise BP at a given submaximal intensity. Despite the novel and interesting data from this previous study [13], the relationship between fitness, movement behaviors, and exercise BP remains poorly understood, particularly in older adults. More information about the complex interplay between these aspects could provide valuable insights on potential targets to attenuate BP response to submaximal efforts, which has been recognized as a risk factor for adverse CVD-related events and mortality [4,5].

Recent theoretical models have proposed network analysis as a robust method for examining complex systems [14,15]. Network analysis is a highly suitable methodological framework for exploring the complex and multidimensional associations between fitness, movement behaviors, and exercise BP. This approach provides a multivariate platform that allows for the simultaneous evaluation of direct and indirect associations among variables within this complex system [16]. Network analysis can help disentangle these relationships, identify potential intervention targets, and highlight mediating factors, thereby enhancing our understanding of underlying mechanisms. The strength of network analysis lies in its ability to model associations while accounting for multicollinearity among predictors [17], a common challenge using traditional analyses. Furthermore, network analysis can incorporate various confounding factors that influence observed associations. Including these covariates ensures the findings reflect true relationships rather than artifacts of unaccounted variability [16,17]. Therefore, this study aims to investigate the associations between fitness and movement behavior components with exercise BP in older adults using a network perspective. This approach may offer insights into the understanding of direct and indirect associations between fitness and movement behaviors with exercise BP in this population, with the potential to suggest pathways for further investigations.

## Methods

### Study design

This cross-sectional, exploratory study was conducted in accordance with the Declaration of Helsinki at Onofre Lopes University Hospital in Natal, Brazil, between June 2018 and December 2019. The study adhered to the Strengthening the Reporting of Observational Studies in Epidemiology (STROBE) guidelines [18] and was approved by the Research Ethics Board of Onofre Lopes University Hospital (protocol CAAE: 82609318.0.0000.5292). All participants were informed about the study procedures and provided written informed consent upon arrival for data collection.

### Participants

Community-dwelling older adults, aged 60–80 years, participated in this study. We employed an open, non-probabilistic community-based recruitment strategy, participants were recruited through electronic flyers on social media, healthcare facilities, community centers for older adults, radio advertisements, and television broadcasts. The eligibility criteria were as follows: (i) no prior history of major adverse cardiovascular events (MACEs) and diagnosed CVD; (ii) no significant muscle, joint, or bone limitations that would restrict their ability to engage in physical exercise; (iii) no recent decompensation due to diabetes or hypertension (i.e., glycemia ≥ 300 mg/dL; resting BP ≥ 160/105 mmHg). More details are available elsewhere [19,20].

### Methodological framework

The methodological framework was designed to explore the associations between fitness and movement behaviors with immediate post-exercise BP in older adults using a network perspective. The primary outcome was immediate post-exercise BP, as a proxy of exercise BP response to light-moderate activities. Exposures included fitness measures (six-minute walk test, handgrip strength, and 30-second chair sit-to-stand test) and accelerometer-measured movement behaviors (moderate-to-vigorous physical activity and sedentary time), both linked to MACEs and CVD in older adults [21–23]. These factors may also be associated with lower exercise BP [10–12]. Additionally, we accounted for traditional CVD risk factors that may have direct or indirect associations with fitness, moderate-to-vigorous physical activity (MVPA), sedentary time, and exercise BP, including age, sex (reference group = females), body mass index (BMI), resting BP, and use of antihypertensive medication (reference group = users of antihypertensive medication) [12].

### Blood pressure measurement and exercise test

All measurements were conducted in a controlled laboratory setting. Upon arrival, participants first underwent a seated resting BP assessment, following a 10-minute rest period. Three or more BP readings were obtained using a validated oscillometric device, with 1-minute intervals between them, until a difference equal to or less than 4 mmHg in SBP or 2 mmHg in DBP was found. The average of the last two measurements was used for analysis [24].

Next, All participants performed a 3-minute moderate walking on a treadmill (Movement, RT150 G3, Pompéia, Brazil) at a speed of 2.7 km/h with a 10% grade (5 METs, equivalent to the first stage of the Bruce protocol). Following established recommendations [10], we implemented a brief, submaximal, and fixed-intensity exercise protocol to assess the exercise BP response to the same motor task for all participants. This approach allows us to assess BP response to a specific external load – in this case, a brief, low-speed walk with a grade.

Immediately (<30 seconds) after test cessation, participants were instructed to sit down to measure immediate post-exercise systolic BP, a proxy of exercise BP. Blood pressure was measured at rest and immediately post-exercise using a validated oscillometric device (Omron, HEM-780-E, Kyoto, Japan) [25], with participants seated in a chair approximately one meter from the treadmill. The same protocol was previously used by our group [20].

Following the cardiovascular assessment, participants completed three physical fitness tests: the six-minute walk test (6MWT), handgrip strength (HGS), and the 30-second sit-to-stand test (30-sSST). All tests followed standard protocols [26–28].

### Fitness

Fitness components commonly used in clinical practice were included, as proxies of cardiorespiratory fitness (six-minute walk test) and muscle strength (30-second sit-to-stand test and handgrip strength) [26,27]. Regarding six-minute walk test (6MWT), all participants were instructed to walk the maximum possible distance in a covered rectangular area measuring 20m x 10m. The total distance walked was recorded. For 30-second sit-to-stand test (30-sSST), all participants were instructed to sit down and stand up from a chair as many times as possible within 30 seconds. The number of repetitions was recorded [26]. Finally, for handgrip strength (HGS) test, all participants were asked to squeeze the dynamometer (Jamar, 5030J1, Chicago, IL, USA) as hard as possible with one hand while seated, with the elbow flexed at 90°. Each hand was tested three times, alternating hands between attempts, with a 60-second rest between measurements. The maximal value, considering both hands, was considered for data analysis [28].

### Accelerometer-measured movement behaviors

Following the laboratory session, participants were instructed to wear a triaxial accelerometer (ActiGraph GT3X) on the right hip for seven consecutive days, during both waking and sleeping periods, excluding water-based activities. (e.g., showering and swimming). Moderate-to-vigorous physical activity and sedentary time were objectively measured using an ActiGraph GT3X accelerometer (ActiGraph LLC, Pensacola, FL, USA). Only participants with at least three valid weekdays (i.e., ≥ 10 hours/day of wear time during waking periods) and at least one valid weekend day were included in the data analysis [29]. Accelerometer non-wear time was defined as ≥90 minutes of zero counts, with a tolerance of up to 2 minutes of ≥100 counts per minute (cpm) [30]. The cut-offs used to define sedentary time and MVPA were: 0–99 cpm and ≥1952 cpm, respectively [31,32]. Each participant received an individual logbook to document their waking and sleeping hours, following the recommended accelerometry procedures [33]. This logbook data was used to establish the accelerometer wear time, specifically during waking hours for each participant. According to the recommendations, the validity of a day is not affected by the duration of sleep [33]. Additionally, participants recorded non-wear time during waking hours when the accelerometer was removed (e.g., during showering). Data were processed using the ActiLife version 6.13.3.2 software program.

### Covariates

Additional information was obtained through face-to-face interviews, including age (in years), self-identified ethnicity (categorized as pardo/black or white), smoking history, educational background (post-secondary education), and medical diagnosis of hypertension with a current antihypertensive medication use. Body weight (in kilograms, Welmy, W300, Brazil) and height (in meters, Welmy, W300, Brazil) were measured, and body mass index (BMI) was calculated as weight in kilograms divided by the square of height in meters (kg/m^2^).

### Statistical analysis

Continuous data are expressed as mean ± standard deviation (SD), and categorical data as absolute and relative frequencies. A network analysis was conducted to examine the associations between fitness and movement behaviors with immediate post-exercise BP. We employed partial correlation as the estimation method for our network analysis, rather than regularized techniques such as EBICglasso, due to the conceptual aim of this study and the nature of our variables. Given the high theoretical and statistical interdependence between resting and immediate post-exercise blood pressure, the use of EBICglasso, which applies penalization to reduce spurious connections, would disproportionately shrink weaker but potentially meaningful associations, especially indirect paths mediated by resting BP [16,34]. This could mask important patterns in the complex interplay between fitness, physical activity, and cardiovascular outcomes. Partial correlation, by contrast, allows for the preservation of all conditional associations between variables, providing a more transparent view of both direct and indirect connections, which aligns with our exploratory, system-oriented objective [16]. Furthermore, previous literature supports the use of partial correlation in network modeling when the goal is to identify nuanced interrelations without over-simplifying the structure of the network, particularly in studies with modest sample sizes and high multicollinearity among predictors [16,34]. Although obtained through different statistical methods, partial correlations are closely related to coefficients obtained in multiple regression models [16]. Therefore, associations with r > 0.1 were considered meaningful [35].

This network analysis, derived from graph theory, consists of elements called nodes (variables) and edges (associations between variables), where red colors indicate negative associations and blue colors indicate positive associations. The thickness and intensity of the color represent the strength of these associations. The “Fruchterman–Reingold” algorithm was applied, positioning data in a relative space such that variables with stronger associations are placed together, while those with weaker associations are repelled from each other [36].

Centrality indicators were reported. Each of these indices was normalized (mean = 0, SD = 1), with an index value >1 indicating a value greater than 1 SD from the mean. Betweenness is estimated based on the number of times a node is part of the shortest path between other pairs of nodes [37]. Closeness refers to the degree to which a node is closely connected to other nodes through the shortest paths [38]. Higher values suggest that the variable can spread the effect of an intervention more quickly. The strength indicator, which is the sum of the weights of all paths connecting a node to others, is crucial for identifying variables with the most robust connections in the network. The expected influence identifies variables that are more sensitive to interventions due to having more connections with other network variables [39].

To assess the robustness of the network and the centrality measures, both accuracy and stability of the network were evaluated. The accuracy of the edges was assessed using a bootstrap method, which generates confidence intervals for the edges, allowing the estimation of the precision of associations between nodes. Edges with wide intervals indicate lower confidence in the strength of the connection. The stability of the centrality measures was examined using the case-dropping bootstrap method, which calculates the Correlation Stability Coefficient (CS-coefficient). This index indicates the maximum proportion of cases that can be dropped from the sample without compromising the consistency of the centrality estimates. A CS value above 0.25 is considered acceptable [34]. Additional subgroup analyses were conducted to address potential differences in immediate post-exercise systolic BP related to sex and antihypertensive medication use. Mean values were compared between males and females, and between users and non-users of antihypertensive medications. Analysis of covariance (ANCOVA) was used to adjust for age and BMI (See S1 Table). All analyses were conducted using RStudio software version 4.4.1 (Posit, USA) with the bootnet, ggplot2, and qgraph packages. Network plot was redrawn using JASP version 0.18.3 [16].

## Results

**Fig 1:**
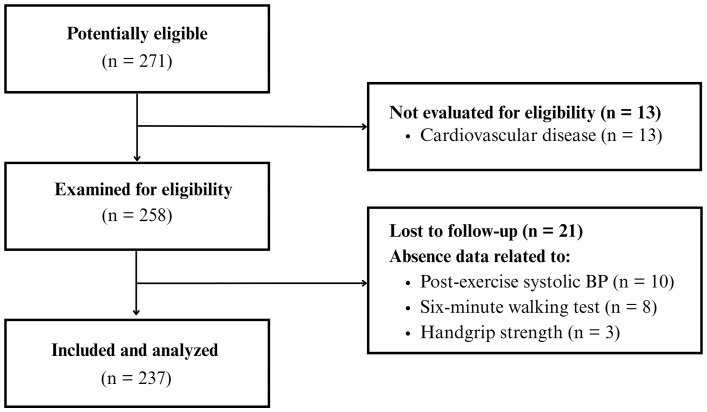
Flowchart of the study. Abbreviations: BP, blood pressure.

A total of 237 older adults were included in the study (see Fig 1). Table 1 displays the participants’ characteristics. The mean age and BMI of participants was 65.8 years and 28.8 kg/m^2^, respectively. Most of them were females (78.1%) with a self-declared skin color Pardo or Black (62%), and approximately one out of four participants had post-secondary education. Regarding BP status, 62% were currently taking antihypertensive medications, most of them under monotherapy (50.2%). The mean absolute immediate post-exercise BP was 160 mmHg, with a mean delta of change of 34 mmHg. Regarding fitness, the mean values of 6MWT, 30-sSST, and HGS were 493 meters, 13 repetitions, and 28 kgf, respectively. Finally, on average, participants spent 21.6 min/day of MVPA and 10.5 h/day in sedentary time.

**Table 1:** Participants’ characteristics (*n* = 237).

	Mean ± SD or n (%)
Age, years	65.8 ± 4.9
Females, n (%)	185 (78.1)
Body mass index, kg/m^2^	28.8 ± 4.6
Post-secondary education, n (%)	55 (23.2)
Skin color, n (%)	
*Pardo*/Black	147 (62)
White	90 (38)
Antihypertensive medication, n (%)	147 (62.0)
Monotherapy	74 (50.2)
Combination therapy	73 (49.8)
Calcium channel blockers	15 (10.2)
Diuretics	55 (37.4)
Angiotensin II receptor blockers	103 (70.0)
ACE inhibitors	7 (4.7)
Beta-blockers	58 (39.5)
Resting systolic BP, mmHg	127 ± 16
Resting diastolic BP, mmHg	71 ± 9
Immediate post-exercise systolic BP, mmHg	160 ± 24
Delta of change in post-exercise systolic BP, mmHg	34 ± 18
Health-related fitness	
Six-minute walk test, m	493 ± 74
Handgrip strength, kgf	28.3 ± 7.6
30-second sit-to-stand, repetitions	13.2 ± 3.1
Accelerometer-measured movement behaviors	
Moderate-to-vigorous physical activity, min/day	21.6 ± 20.8
Sedentary time, h/day	10.5 ± 1.6

Data are shown as mean ± SD or absolute (n) and relative (%) frequencies. Abbreviations: ACE, angiotensin-converting-enzyme; BP, blood pressure.

Among participants taking antihypertensive medication (n = 147), 104 (77.6%) were female and 30 (22.4%) were male, while for participants not taking antihypertensive medication (n = 103), 81 (78,6%) were female and 22 (21,4%) we male reflecting the overall sex distribution of the sample. The mean distance covered in the six-minute walking test was 533 ± 82 meters for males and 481 ± for females. We conducted additional descriptive analyses by subgroup to explore immediate post-exercise systolic blood pressure. Males exhibited slightly higher immediate post-exercise systolic BP values compared to females (167 ± 26 mmHg vs. 157 ± 23 mmHg), and individuals using antihypertensive medications presented higher values than non-users (163 ± 24 mmHg vs. 155 ± 24 mmHg). Supplementary analyses comparing immediate post-exercise systolic BP across subgroups revealed no statistically significant differences between males and females, nor between antihypertensive medication users and non-users, after adjusting for age and BMI using ANCOVA (p > 0.05 for all comparisons). While males and medication users presented slightly higher mean post-exercise systolic BP values, these trends did not reach significance (see S1 Table).

**Fig 2:**
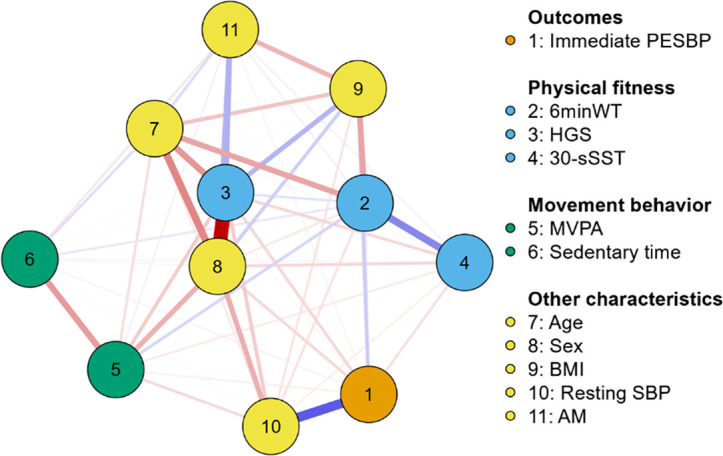
Network topology of fitness, movement behaviors, and immediate post-exercise systolic blood pressure in older adults (n = 237). Nodes represent individual variables, and edges represent partial correlations between them. Blue edges indicate positive associations, while red edges indicate negative associations. The thickness and intensity of each edge reflect the strength of the association (i.e., higher weights yield thicker and more saturated lines). All edges represent direct associations after controlling for all other variables in the network. The Fruchterman–Reingold algorithm was used to position nodes such that strongly connected variables appear closer together. Abbreviations: 1. Immediate PESBP, immediate post-exercise systolic blood pressure (mmHg); 2. 6MWT, six-minute walk test in meters; 3. HGS, handgrip strength test in kgf; 4. 30-sSST, 30-second sit-to-stand test in repetitions; 5. MVPA, moderate-to-vigorous physical activity in min/day; 6. Sedentary time, in h/day; 7. Age, in years; 8. Sex, reference group being females; 9. BMI, body mass index in kg/m2; 10. Resting SBP, resting systolic blood pressure in mmHg; 11. AM, antihypertensive medication, reference group being taking antihypertensive medication.

Network accuracy and stability of the centrality metrics are presented on S1 and S2 Figs, respectively. All edges remained within the confidence interval, indicating the robustness of the network. The CS-coefficient remained above 0.25 throughout the analysis, indicating the consistency of the centrality estimates. Fig 2 and Table 2 display the individuals associations of each variable. The first column of the weight matrix (Table 2) presents the associations between immediate post-exercise systolic BP with each variable (i.e., fitness, movement behaviors, and the other participants’ characteristics).

**Table 2:** Weight matrix of associations between fitness and movement behaviors with immediate post-exercise systolic blood pressure in older adults (n = 237).

Variable	1	2	3	4	5	6	7	8	9	10	11
PESBP (1)	–										
**Fitness**											
6MWT (2)	0.074	–									
HGS (3)	−0.136	0.098	–								
30-sSST (4)	−0.106	0.423	−0.138	–							
**Movement behaviors**											
MVPA (5)	−0.053	0.142	−0.175	−0.071	–						
Sedentary time (6)	0.038	0.082	−0.019	0.032	−0.349	–					
**Other characteristics**											
Age (7)	−0.029	−0.315	−0.362	0.021	−0.112	0.110	–				
Sex (8)	−0.131	−0.076	−0.898	−0.137	−0.274	−0.034	−0.427	–			
BMI (9)	0.181	−0.323	0.260	−0.002	−0.011	−0.016	−0.205	0.169	–		
Resting SBP (10)	0.581	−0.099	−0.176	−0.055	−0.128	0.016	−0.075	−0.281	−0.091	–	
AM (11)	−0.039	0.048	0.282	0.046	0.048	−0.084	0.120	0.263	−0.257	−0.007	–

Abbreviations: AM, antihypertensive medication; BMI, body mass index; HGS, handgrip strength; MVPA, moderate-to-vigorous physical activity; PESBP, post-exercise systolic blood pressure; SBP, systolic blood pressure; 6MWT, six-minute walk test; 30-sSST, 30-seconds sit-to-stand test.

Higher body mass index (BMI) showed a moderate direct association with immediate post-exercise systolic BP (weight: 0.181), indicating a contribution of increased BMI to a higher immediate post-exercise systolic BP. Lower handgrip strength (weight: −0.136) and fewer repetitions in the 30-second sit-to-stand test (weight: −0.106) were directly associated with higher immediate post-exercise systolic BP, indicating relevant links between reduced muscular strength and cardiovascular response. Although these coefficients are smaller than that of resting BP (weight: 0.581), they represent additional variables with meaningful associations in the network structure.

Lower performance in the six-minute walk test (weight: −0.099), lower handgrip strength (weight: −0.176), and reduced time spent in moderate-to-vigorous physical activity (weight: −0.128), were associated with higher resting BP, indicating relevant links between overall fitness and physical activity to cardiovascular response, which in turn are indirectly associated with immediate post-exercise systolic BP via resting BP. These variables demonstrated relevant indirect contributions within the network model. Also, male sex (reference = female) was directly associated (weight: −0.131) and indirectly associated via resting BP (weight: −0.281) with higher post-exercise BP.

Centrality values from the network analysis are presented in Table 3. Regarding fitness components and movement behaviors, 6MWT, 30-sSST and sedentary time showed meaningful values of expected influence, indicating them as highly interconnected within the network. Betweenness centrality indicates MVPA as a variable capable of mediating other connections within the network. High closeness and strength indicators were observed for HGS, suggesting that this variable is capable of rapidly propagating its influence, either positive or negative, to other components of the network. Additionally, centrality indicators highlighted importance of immediate post-exercise systolic BP, antihypertensive medication, age, sex and resting systolic BP within the network.

**Table 3:** Network centrality indicators for each variable.

Variables	Betweenness	Closeness	Strength	Expected influence
PESBP	−0.594	−0.750	−0.373	**1.162**
**Fitness**				
6MWT	0.059	0.027	0.162	**0.723**
HGS	−0.267	**1.347**	**1.643**	−1.052
30-sSST	−0.758	−0.983	−0.951	**0.668**
**Movement behaviors**				
MVPA	**0.713**	−0.267	−0.385	−0.673
Sedentary time	−0.758	−1.472	−1.382	**0.350**
**Other characteristics**				
Age	−0.267	**0.762**	**0.325**	−1.065
Sex	**2.674**	**1.867**	**1.892**	−1.809
BMI	−0.267	0.238	−0.126	0.255
Resting SBP	**0.223**	−0.375	−0.131	0.227
AM	−0.758	−0.393	−0.674	**1.215**

Abbreviations: AM, antihypertensive medication; BMI, body mass index; HGS, handgrip strength; MVPA, moderate-to-vigorous physical activity; PESBP, post-exercise systolic blood pressure; SBP, systolic blood pressure; 6MWT, six-minute walk test; 30-sSST, 30-seconds sit-to-stand test.

## Discussion

To the best of our knowledge, this is the first study to explore the associations between fitness and movement behaviors with immediate post-exercise BP using a network analysis in older adults. Our main findings demonstrated that: (i) lower HGS and 30-sSST performances were directly associated with a higher immediate post-exercise BP response; (ii) lower 6MWT, HGS, and MVPA were indirectly associated with higher immediate post-exercise BP response via resting systolic BP; (iii) centrality indicators highlighted 6MWT, 30-sSST and sedentary time as potential targets for interventions; MVPA as a central hub, mediating connections within the network; HGS as a variable capable of rapidly propagating its influence to other components of the network. Taken together, our findings provide new insights into the potential direct and indirect roles of fitness and movement behaviors on immediate post-exercise systolic BP, a proxy for exercise BP, in older adults.

In our first-step analytical approach, the weight matrix of associations demonstrated that HGS (weight: −0.136) and 30-sSST (weight: −0.106), but not 6MWT, had meaningful direct associations with immediate post-exercise BP. This finding may suggest that individuals with lower muscle strength may experience higher cardiovascular overload, including higher BP response, during submaximal motor tasks that require both aerobic and strength-based efforts. This appears to be the case in our study, which involved a standardized, brief, low speed walking at 2.7 km/h with a 10% incline, like uphill walking in daily life. To support our findings, Lovell et al. [40] demonstrated that 16 weeks of resistance training increased muscle strength and reduced BP response to submaximal exercise (at 40 watts and 50% of maximal oxygen uptake) in older adults.

In contrast to our findings, previous studies have demonstrated that higher cardiorespiratory fitness is directly associated with lower exercise BP during submaximal efforts [12,41,42]. However, there are important methodological differences that should be highlighted. None of the previous studies focused exclusively on older adults; they included various populations, such as adolescents and general adults, with mixed samples of young adults, middle-aged individuals, and older people [12,41,42]. Muscle strength was not accounted for in any of these studies’ models; supervised submaximal or maximal incremental exercise testing was included to assess cardiorespiratory fitness, which differs from the 6MWT, where participants self-select the walking intensity. Importantly, the 6MWT is well-recommended for clinical purposes and serves as a valid proxy for cardiorespiratory fitness, with a moderate-to-strong correlation to time to exhaustion (r = 0.78) and peak oxygen uptake (r = 0.76) [43,44].

Lower performances on 6MWT (weight: −0.099) and HGS (weight: −0.176), and lower levels of MVPA (weight: −0.128) were significantly associated with higher resting systolic BP, which had the strongest association with immediate post-exercise BP (weight: 0.581). These findings suggest an indirect association between the above-mentioned fitness and movement behavior components with exercise BP via resting BP, i.e., these factors may contribute to lower resting BP levels, which ultimately is associated with an attenuated exercise BP response. Also, resting systolic BP exhibited meaningful betweenness centrality values (0.223), further supporting its mediating role between fitness and movement behaviors on immediate post-exercise BP. Our findings are in accordance with Moore et. al. [12], who found that all CVD risk factors, except age, had a pathway of association with exercise BP indirectly via resting BP.

In our study, no direct associations were found between immediate post-exercise BP and either MVPA or sedentary time. This finding contrasts with previous evidence suggesting that MVPA contributes to improved cardiovascular responses during and after exercise [40]. However, our network analysis highlighted that MVPA was indirectly associated with immediate post-exercise BP via its association with resting systolic BP. This may suggest that individuals with higher MVPA levels and lower sedentary time may have lower resting BP, which, in turn, could contribute to a more favorable BP response to exercise. Nonetheless, the absence of a direct link between MVPA and sedentary time with immediate post-exercise BP underscores the complexity of these interactions and the potential role of other intrinsic physiological and vascular factors, such as vascular stiffness [20] and arterial compliance [45], that were not directly assessed in our study.

Regarding fitness and movement behaviors, the expected influence indicator highlighted the 6MWT (0.723), 30-sSST (0.668), and sedentary time (0.350) as variables that are more connected with other network variables, indicating them as potential targets for interventions [39]. The betweenness indicator underscores MVPA (0.713) as a variable capable of mediating associations between different pairs of nodes within the network, acting as a central hub within the network [37]. From a rational standpoint, our findings are logically supported, as improvements in cardiorespiratory fitness and muscular strength, along with reductions in sedentary time, are statistically linked to increased levels of MVPA, which emerged as a central node mediating several associations in the network. Furthermore, HGS exhibited high strength (1.643) and closeness (1.347) centrality, indicating its capacity to rapidly propagate the effect of an intervention to other components of the network [38]. These findings indicate that muscular strength is associated with multiple variables within the network, and its enhancement could coincide with broader shifts in the system. Accumulating evidence supports the inverse association between HGS with CVD risk and mortality in older adults [22,46]. Importantly, while centrality metrics highlight key nodes within the system, they do not pinpoint the specific causal pathways or mechanisms through which these benefits propagate [16,17].

From a clinical perspective, measuring BP during exercise presents significant technical challenges, such as excessive arm movements and noise from treadmills, which make it unfeasible in some clinical settings. In addition, exercise BP cannot be assessed during some field tests commonly used in clinical practice, such as the 6MWT. An alternative approach is to measure BP immediately after exercise [5], as done in our study. This approach may offer some advantages, including a stable position for participants and reduced interference from noise and movement. Additionally, it allows the use of automated oscillometric devices [20,45]. However, it is important to note that immediate post-exercise BP may reflect a distinct physiological phenomenon, as it also captures the early phase of the effort-recovery transition. Despite this, previous studies have shown a significant association between immediate post-exercise BP with impairments on vascular function [20,45], suggesting potential for clinical purposes.

Some physiological aspects may partially explain our data, suggesting that muscle strength is associated with lower exercise BP in older adults, potentially reflecting a physiological link worth further investigation. A previous study [47] suggested that older adults with low muscle strength (dynapenia) may have an impaired metaboreflex control, resulting in reduced muscle perfusion during metaboreflex activation compared to peers with similar muscle mass but without dynapenia. Thus, during uphill walking, older adults with lower muscle strength may have an impaired ability to redistribute blood flow to active muscles, leading to a greater compensatory increase in BP (i.e., a higher exercise BP response). Another potential explanation is that uphill walking requires significant activation of the quadriceps, a muscle group that typically experiences an earlier decline in strength and mass compared to other lower-limb muscles [48,49]. Additionally, although aging is associated with a reduction in the proportion and size of type II muscle fibers, the quadriceps retains a relevant functional presence of these fibers. Given their higher glycolytic profile and greater susceptibility to metabolite accumulation, type II fibres may disproportionately drive the pressor response during uphill walking, particularly in older adults with reduced muscle strength. This aspect may also partially explain the higher muscle sympathetic nervous activity mediated by the metaboreflex in muscle groups with a higher proportion of type II fibers [50].

Regarding other participants’ characteristics, higher BMI and male sex were associated with higher immediate post-exercise BP, which aligns with previous findings [12,41,42]. Evidence suggests that males [51] and individuals with higher BMI [52] have increased vascular resistance and autonomic nervous activity, which may explain the increased exercise BP. Immediate post-exercise BP and antihypertensive treatment exhibited high expected influence values, suggesting a substantial direct or indirect association with fitness, movement behaviors, and other network components. Age and sex high centrality indicators reflect age- and sex-related impact on BP regulation [51], fitness [53], and movement behaviors [54].

Despite the novel findings, our study has limitations that should be mentioned. First, as this study is a secondary analysis not originally designed for this specific purpose, we did not assess heart rate (HR) responses or peak BP during the exercise test. This hinders our ability to determine the HR zones reached by participants during testing, determining the percentage of maximal HR achieved during the task would have offered useful insights into interindividual variability in exertion and cardiovascular response, which could provide a better understanding about the intensity reached by them. However, in our study, the central objective was to evaluate the systolic BP response to a fixed and standardized submaximal motor task—a 3-minute walk at 2.7 km/h with a 10% incline—which simulates a common functional challenge in daily life for older adults. This design intentionally prioritized the BP response to a controlled workload over relative effort levels such as percentage of age-predicted maximal HR. In addition, it was not possible to compare immediate post-exercise systolic BP with peak exercise BP, i.e., it remains unclear if these measures are highly correlated or not. Second, immediate post-exercise BP measurement timing was not standardized across participants due to individual differences in mobility and cuff inflation time which may have influenced post-exercise cardiovascular recovery. This methodological approach was chosen to address situations where it is not feasible to measure BP during exercise. Accordingly, we referred to this variable as “immediate post-exercise BP” rather than “exercise BP,” which is in line with current recommendations [5]. The variability introduced by non-standardized timing can mask an elevated BP response if there is a delay in measurement, especially in people with better physical conditioning, who tend to have a faster drop in BP after physical exertion. However this variability likely reflects real-world conditions, and the consistent associations observed across the sample suggest that this limitation does not significantly compromise the robustness of the study’s findings. Third, the sample was predominantly composed of women (78%) and antihypertensive medication users (62.0%), which reflects common trends in voluntary participation in aging and physical activity studies in our country [55,56]. While this demographic pattern may limit the generalizability of our findings to more balanced populations, both sex and medication use were explicitly included as covariates in our network model. Their respective centrality metrics suggest that their influence was captured within the multivariate structure of associations, supporting the robustness of our findings despite the sample heterogeneity. Future studies with stratified or representative sampling designs may further elucidate sex- and treatment-specific patterns of blood pressure response to exercise. Fourth, although more detailed body composition metrics such as fat percentage, waist circumference, or muscle mass could provide greater physiological specificity, we intentionally employed simple and widely accessible measures, such as BMI and functional strength tests. This choice reflects the pragmatic reality of clinical and community health settings, where more advanced assessments are often unavailable. Likewise, metabolic syndrome was not formally assessed, as its diagnostic criteria require biochemical markers that are often unavailable in routine evaluations performed by non-specialist health professionals. Nonetheless, key clinical components of the syndrome—such as elevated blood pressure and general adiposity—were included in the model via resting BP and BMI, thereby partially accounting for underlying cardiometabolic risk. Fifth, although the difference between resting and immediate post-exercise systolic blood pressure (ΔSBP) may offer additional physiological insights, we deliberately selected the absolute immediate post-exercise systolic BP as the primary outcome. This decision was based on two key considerations: i) absolute immediate post-exercise systolic BP has a broader body of evidence supporting its clinical relevance as a predictor of cardiovascular risk [5,57]; ii) analyzing ΔSBP would preclude statistical adjustment for resting SBP due to collinearity (i.e., mathematical dependency), making it unfeasible to examine indirect associations mediated by resting SBP—an important objective of our network analysis. Additionally, introducing a second network model based on ΔSBP would significantly expand the scope of results and discussion, potentially shifting the focus away from the main outcome. We suggest that future studies may address the role of ΔSBP using complementary analytical approaches such as mediation analysis or growth curve modeling. Despite limitations, the strengths of our study include the objective measurement of movement behaviors using accelerometry, which minimizes biases inherent in self-reported data (recall bias). Also, we included different measures of fitness, allowing a better understanding about some fitness characteristics and their associations with immediate post-exercise BP. Furthermore, network analysis enabled a more nuanced understanding of the complex association between fitness and movement behaviors with immediate post-exercise BP in older adults.

## Conclusion

Our findings indicate a complex interplay — both direct and indirect (via resting BP) — between fitness, movement behaviors, and immediate post-exercise BP in older adults. Higher levels of moderate-to-vigorous physical activity, cardiorespiratory fitness, and muscle strength, along with lower sedentary time, may be associated with reduced immediate post-exercise BP in this population. These associations highlight potential factors of interest for future longitudinal and interventional studies. Moreover, such findings may inform clinical screening and support the design of physical activity programs aimed at improving cardiovascular responses to daily physical tasks in older adults.

## Supporting information

S1 Fig Non-parametric bootstrap analysis with 1000 resamples. Shaded area denotes the confidence interval.
(TIF)

S2 Fig Stability of the centrality metrics. (TIF)

S1 Table Post-exercise systolic blood pressure stratified by sex and antihypertensive medication use. (DOCX)
