# Supplementary figures and images for: Associations between fitness, movement behaviors, and immediate post-exercise blood pressure in older adults: A network perspective
Source: PLoS One. 2025 Jul 30;20(7):e0329280. doi: 10.1371/journal.pone.0329280 (PMC12309990; doi:10.1371/journal.pone.0329280)

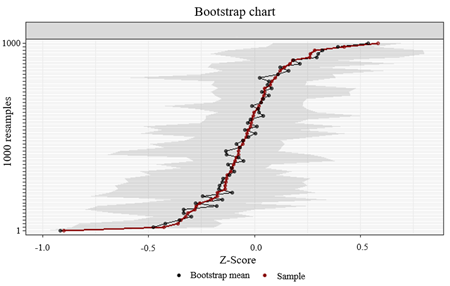

Supplement: S1 Fig — Shaded area denotes the confidence interval. (TIF) [file pone.0329280.s001.tif]

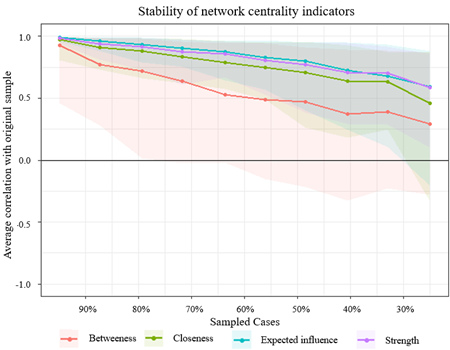

Supplement: S2 Fig — (TIF) [file pone.0329280.s002.tif]
