# Supplementary material for: Associations between fitness, movement behaviors, and immediate post-exercise blood pressure in older adults: A network perspective
Source: PLoS One. 2025 Jul 30;20(7):e0329280. doi: 10.1371/journal.pone.0329280 (PMC12309990; doi:10.1371/journal.pone.0329280)
Supplement: S1 Table — (DOCX) [file pone.0329280.s003.docx]

|  |  | Post-exercise systolic BP (mmHg) | | |
| --- | --- | --- | --- | --- |
|  |  | Sex | | p |
|  |  | Males | Females |  |
| Antihypertensive medication use | Yes | 171 ± 26 (n=30) | 161 ± 22 (n=104) | 0.213 |
|  | No | 161 ± 26 (n=22) | 153 ± 23 (n=81) | 1.000 |
| p | | 1.000 | 0.419 |  |

**Supplementary Table S1.** Post-exercise systolic blood pressure stratified by sex and antihypertensive medication use.

Values represent mean ± standard deviation of immediate post-exercise systolic blood pressure (mmHg). Group comparisons were conducted using analysis of covariance (ANCOVA), adjusting for age and BMI. No statistically significant differences were observed between sexes or medication use groups after adjustment (p > 0.05).
